# Prognosis and Characteristics of Hypermucoviscous Klebsiella pneumoniae Infection in Critically Ill Patients: A Case Series

**DOI:** 10.7759/cureus.59094

**Published:** 2024-04-26

**Authors:** Daichi Yomogida, Hiroyuki Kuwano, Tatsuya Miyakoshi, Shiori Mizuta, Shinjiro Horikawa, Yosinao Koshida

**Affiliations:** 1 Intensive Care Medicine, Toyama Prefectural Central Hospital, Toyama, JPN

**Keywords:** string test, klebsiella pneumoniae (kp), hypermucoviscosity, critical care medicine, antibiotic resistance, antibiotics

## Abstract

Introduction

Hypermucoviscous *Klebsiella pneumoniae* (hvKP) is related to invasive infections; however, there have been very few comprehensive reports on the clinical features and prognosis of critically ill patients with the infection.

Methods

We conducted a retrospective case series in a general intensive care unit in Japan. Patients with positive blood cultures for KP between January 1, 2020 and December 31, 2022 were included. hvKP was defined by the positivity in the string test. We analyzed the patient’s characteristics at baseline, including comorbidities, abscess formation, Sequential Organ Failure Assessment (SOFA) score, Acute Physiology and Chronic Health Evaluation (APACHE) II score, septic shock, duration of hospitalization, 30-day mortality, and infection site.

Results

A total of 24 patients had a positive blood culture for KP; nine patients (37.5%) were positive for the string test (hvKP) while 15 (62.5%) were negative (non-hvKP). In both groups, the patients were old (mean age, hvKP 80.4 vs. non-hvKP 75.7 years) and more often male (five patients (55.6%) vs. 12 patients (80.0%)). No statistically significant difference was found between the two groups in terms of comorbidities, such as diabetes mellitus, chronic obstructive pulmonary disease, chronic kidney disease, and malignancy. No statistical difference was seen in abscess formation (two patients [22.2%] vs. one patient (6.7%)), SOFA score (5.2±4.8 vs. 4.7±3.4), APACHE II score (19.6 (15.0-20.0) vs. 17.0 (11.2-20.8)), septic shock (five patients (55.6%) vs. four patient (26.7%)), duration of hospitalization (37.2 (12.0-51.0) vs. 32.3 (9.5-21.0)), and 30-day mortality (two patients (22.2%) vs. two patients (13.3%)). Two cases with hvKP died within 24 h. No significant difference was seen in the infection sources; respiratory infection (2 (22.2%) vs. 1 (6.7%)), hepatobiliary infection (2 (22.2%) vs. 7 (46.7%)), and genitourinary infection (1 (11.1%) vs. 5 (33.3%)).

Conclusions

Critically ill patients with hvKP infection showed characteristics similar to those reported previously. However, the disease could rapidly become severe and have a poor prognostic outcome.

## Introduction

*Klebsiella pneumoniae* (KP) is a gram-negative lactose-fermenting non-motile aerobic rod-shaped bacterium detected in the stool (5%-38%) and nasopharynx (1%-6%) samples [1]. The hypermucoviscosity phenotype is a major virulence factor of KP. A hypermucoviscous (hv) KP produces a mucoviscous exopolysaccharide web and is identified using a string test [2]. The phenotype is correlated with high serum resistance and invasive infections, such as liver abscesses, compared to that in non-hvKP [3]. Although hvKP is an important causative organism in severe invasive infections, there are only a few comprehensive reports on its clinical features and prognosis of critically ill patients with the infection. Therefore, this study aimed to examine the clinical characteristics and outcomes of patients with hvKP in a cohort admitted to the intensive care unit.

## Materials and methods

Subjects

We performed a retrospective case series in the general intensive care unit (ICU) of a hospital in Japan. All patients were Japanese. This case series study included patients admitted to the ICU with a positive blood culture for KP between January 1, 2020 and December 31, 2022. Patients under 18 years old were excluded. Finally, 24 patients were included. The cases were identified based on positive blood culture results retrieved from medical microbiology databases.

The Medical Ethics Review Committee waived the need for patients’ consent owing to the retrospective nature of this study, in which the participants were not subjected to any form of action. Data are presented according to the reporting guidelines for case series [3].

Methods

**Figure 1:**
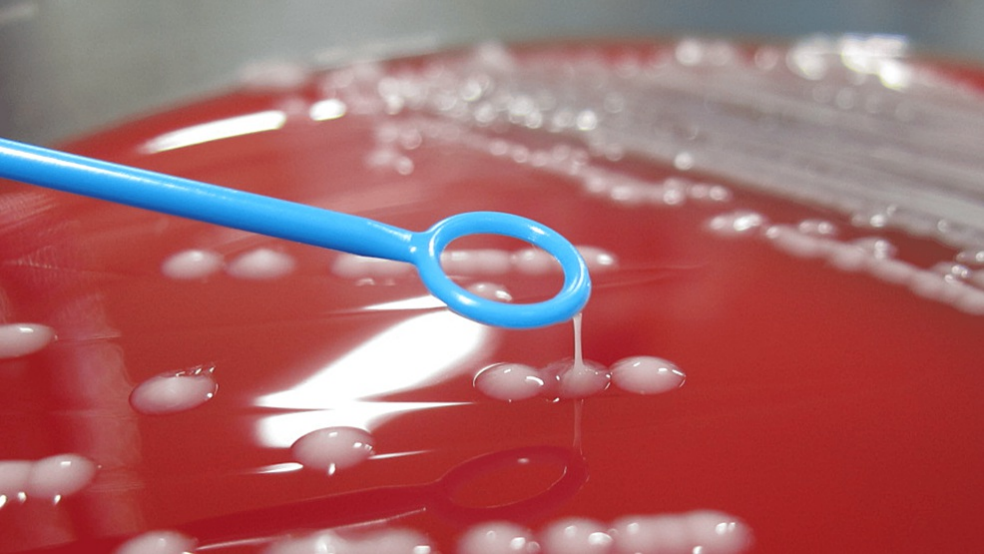
Positive string test result Hypermucoviscous *K. pneumoniae* grows as sticky colonies on agar plates. A positive string test is indicated by a > 5-mm viscous string from the colony on an agar plate when stretched by a standard bacteriologic loop.

The variables include age, sex, comorbidities on admission, abscess formation, Sequential Organ Failure Assessment (SOFA) score, Acute Physiology and Chronic Health Evaluation II (APACHE II) score, septic shock, duration of hospitalization, 30-day mortality, and infectious source. Hypermucoviscosity was defined by a positive string test result (Figure 1).

Bacterial colonies grown overnight on a blood agar plate at 37 °C were stretched by a standard bacteriological loop. If a mucoviscous string > 5 mm in length was formed, the string test was considered positive, and the isolate was identified as hvKP [4].

Statistical analysis

Statistical analyses were performed in November 2023 using R, version 4.3.1 (The R Foundation for Statistical Computing, Vienna, Austria). Differences in continuous variables between the groups were examined using an unpaired Student’s t-test after a symmetrical distribution was confirmed. Otherwise, the Mann-Whitney U test was used. Fisher’s exact test was used to analyze the frequencies of the categorical variables. Patient survival rates were estimated using the Kaplan-Meier method. Statistical significance was set at p < 0.05.

## Results

A total of 24 patients were positive for KP; nine (37.5%) were positive in the string test while 15 (62.5%) were negative. The former group was defined as the hvKP-infected group, and the latter was defined as the non-hvKP-infected group. Blood cultures were collected when the patient visited the emergency department with fever or when the patient developed a fever before ICU admission. In both the groups, patients were aged (mean age, hvKP 80.4 vs. non-hvKP 75.7 years) and more often male (five patients (55.6%) vs. 12 patients (80.0%)). Baseline characteristics of the patients are presented in Table 1.

**Table 1:** Baseline characteristics upon ICU admission Continuous variables with normal distribution are expressed as mean ± SD and variables with non-normal distribution are expressed as median and IQ range (25th and 75th percentiles) CKD; Chronic kidney disease, COPD; Chronic obstructive pulmonary disease, DM; Diabetes mellitus

	hvKP	Non-hvKP	P-value
	（N = 9）	（N = 15）	
Age - years	80.4±5.8	75.7±13.9	0.255
Male - number (%)	5 (55.6)	12 (80.0)	0.3564
DM - number (%)	3 (33.3)	5 (33.3)	1
COPD - number (%)	1 (11.1)	0 (0)	0.375
CKD – number (%)	5 (55.6)	10 (66.7)	0.6785
Malignancy – number (%)	2 (22.2)	2 (13.3)	0.6146

There was no statistically significant difference between the two groups. Upon ICU admission, there was no statistically significant difference in abscess formation, Sequential Organ Failure Assessment (SOFA) score, Acute Physiology and Chronic Health Evaluation II (APACHE II) score, septic shock, or duration of hospitalization (Table 2).

**Table 2:** Severity and prognosis Continuous variables with normal distribution are expressed as mean ± SD and variables with non-normal distribution are expressed as median and IQ range (25th and 75th percentiles) APACHE II; Acute physiology and chronic health evaluation II, SOFA; Sequential organ failure assessment

	hvKP	Non-hvKP	P-value
	（N = 9）	（N = 15）	
Abscess – number (%)	2 (22.2)	1 (6.7)	0.5331
SOFA	5.2±4.8	4.7±3.4	0.769
APACHE II	19.6 (15.0–20.0)	17.0 (11.2–20.8)	0.506
Septic shock - number (%)	5 (55.6)	4 (26.7)	0.2119
Duration of hospitalization – days	37.2 (12.0–51.0)	32.3 (9.5–21.0)	0.59
30-day mortality – number (%)	2 (22.2)	2 (13.3)	0.615

**Figure 2:**
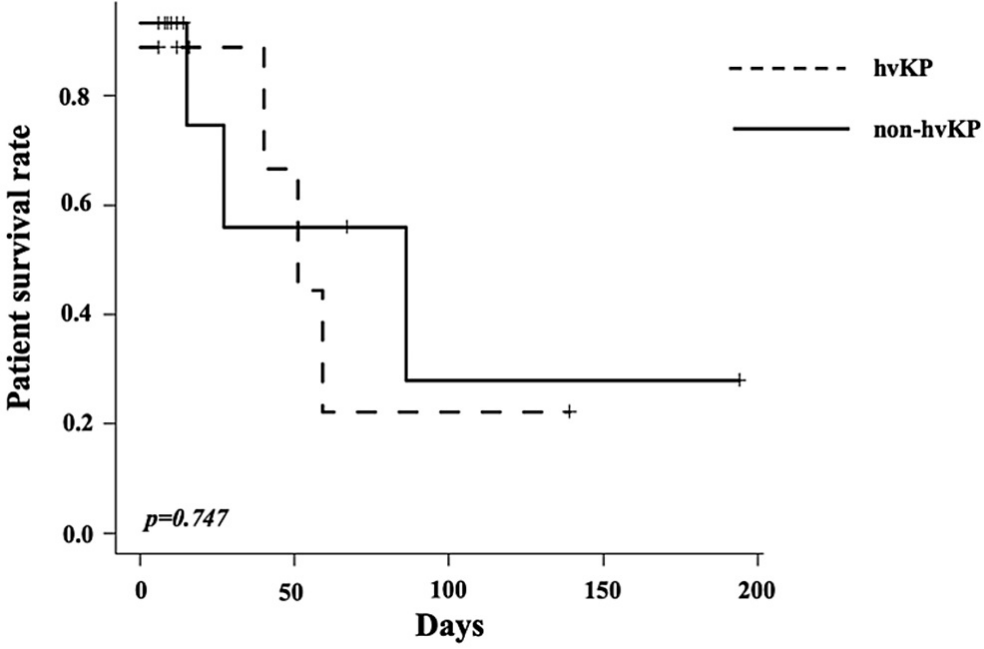
Patient survival curve There was no difference in prognosis between the hvKP group and the non-hvKP group. hvKP; Hypermucoviscous Klebsiella pneumoniae

Further, no difference in life expectancy during hospitalization was seen (Figure 2).

Two patients with hvKP died within 24 h. No significant difference was seen in infectious sources: respiratory infection (2 (22.2%) vs. 1 (6.7%)), hepatobiliary infection (2 (22.2%) vs. 7 (46.7%)), and genitourinary infection (1 (11.1%) vs. 5 (33.3%)). The infectious sources are presented in Table 3. All patients initially received intravenous antibiotic therapy for bacteriological sensitivity.

**Table 3:** Site of infection

	hvKP	Non-hvKP
	（N = 9）	（N = 15）
Respiratory - number (%)	2 (22.2)	1 (6.7)
Hepatobiliary - number (%)	2 (22.2)	7 (46.7)
Genitourinary - number (%)	1 (11.1)	5 (33.3)
Other - number (%)	4 (44.4)	2 (13.3)

## Discussion

To the best of our knowledge, this is one of the first case series of hvKP infection in a critically ill patient. It confirmed the characteristics of hvKP in critical care settings.

KP has five major virulence factors, namely capsular serotype, hypermucoviscosity phenotype, lipopolysaccharide, siderophores, and pili [1]. The mucoviscous exopolysaccharide web produced by hvKP inhibits complement-mediated clearance and is considered a major virulence factor of hvKP [5]. This ability is associated with serum resistance and invasive infections, including abscess formation [6,7]. In a study in Taiwan, of the 51 cases with hvKP, 31 formed abscesses [8]. Based on these features, hypervirulent KP strains are defined as those with a hypermucoviscosity phenotype [9].

The prevalence of hvKP was 37.5% in our study, which is similar to the 38% prevalence reported previously [9]. The rate is generally higher for community-acquired infections than for nosocomial infections. All cases of hvKP were diagnosed as hvKP infection by blood cultures obtained from the emergency department in our study, suggesting that community-acquired infections were common even in critically ill patients.

Several factors that impair host immune responses, such as diabetes mellitus, alcoholism, malignancy, chronic obstructive pulmonary disease, renal failure, and glucocorticoid therapy, have been reported in KP infections [10,11]. However, obvious risks for hvKP infection have not been detected yet, and baseline characteristics are not reported to be different between patients with hv strains and those with non-hv strains [12]. A study on patients with KP liver abscess suggested that diabetes mellitus is a risk factor for hvKP infection; however, some patients developed KP liver abscess without any comorbidity [13]. In our study, the incidence of diabetes mellitus, chronic kidney disease, chronic obstructive pulmonary disease, and malignancy did not differ between the groups. However, in both groups, approximately one-third of the patients had diabetes mellitus, which has been proven to be a risk factor for KP infection, regardless of the hypermucoviscosity phenotype. Specific factors involved in hvKP infection in critically ill patients were not identified in our study, and diabetes mellitus was suggested as a common risk factor for KP infection.

Although hvKP was initially reported as the causative organism of pyogenic liver abscesses [14], previous studies have shown that hvKP can cause disseminated infections as well [15], and the prognosis of hvKP infection is generally worse than that of non-hv phenotypes. Even in patients without comorbidities, the mortality rate is reportedly 3%-42% [16]. In our study, 30-day all-cause mortality was statistically similar in both groups. However, two fatal cases of hvKP died within 24 hours of ICU admission, suggesting that patients with hvKP infection could deteriorate rapidly and have a poorer prognosis. The relationship between SOFA or APACHE II scores and KP infection has not been reported yet. Our current study revealed no statistically significant difference in these scores; however, the number of cases was limited, and the two fatal cases suggested that some cases of hvKP infection were more severe than those of non-hvKP infection. On the other hand, 30-day mortality was not different in our study, which also suggested that mortality can be evitable by an appropriate therapy, including drainage procedures and antibiotic therapies, even in critical cases with hvKP.

In our study, there was no tendency towards the infection site. We focused only on blood culture samples, and all the included patients had disseminated infections. According to previous studies, abscess formation is strongly associated with the hv phenotype [17], and hvKP developed abscesses not only in the liver but also at other sites with the spread of metastasis. In our study, the frequency of abscess formation did not differ between hv and non-hv phenotypes. However, the number of patients was limited, and further analyses would be required in this regard.

Our study had several limitations. First, the retrospective design of our study created a risk of information bias. Although we comprehensively assessed all patient records, possible inaccuracies in the provided data cannot be ruled out. Second, overlapping infections with bacteria other than KP could not be ruled out, because we analyzed only blood cultures. Third, the number of patients included in the study was limited.

## Conclusions

In this case series, we found that critically ill patients with hvKP infection showed characteristics similar to those reported previously, in terms of patient background, comorbidities, and prognosis. In contrast, a review of two fatal cases of hvKP infection showed that the disease could rapidly become severe and have a poor prognostic outcome. Therefore, further case studies and analyses would be required in future.
